# Genes that improved fitness also cost modern humans: evidence for genes with antagonistic effects on longevity and disease

**DOI:** 10.1093/emph/eoz002

**Published:** 2019-01-23

**Authors:** Sean G Byars, Konstantinos Voskarides

**Affiliations:** 1 Melbourne School of Population and Global Health, University of Melbourne, Parkville, Victoria, Australia; 2 Medical School, University of Cyprus, Nicosia, Cyprus

Austad and Hoffmann review the current state-of-the-art on what support there is for the theory of antagonistic pleiotropy and what implications this has for modern medicine regarding improving human health and longevity [1]. Although the authors focus on examples in both wild populations and laboratory conditions, the review states that there are no compelling examples in humans where the underlying genes or alleles that carry this tradeoff have been identified. This fails to acknowledge recent studies, mostly published the last two years, where excellent progress has been made in identifying such genes and below, we describe several examples.

These studies are essential to the discussion on aging biology in general and human health. As a species, we uniquely live well beyond reproductive senescence and thus the potential for costs of such antagonistic variants to express themselves is particularly important to assess. Two studies in 2017 uncovered evidence for antagonistic pleiotropy in genes related to coronary heart disease (CAD) and fitness, and diseases related to ageing.

The study by Byars *et al.* [2], found that CAD genes in humans are significantly enriched for fitness (increased lifetime reproductive success) relative to the rest of the genome, with evidence that the direction of their effects on CAD and fitness are antagonistic. This study provides a possible reason why genes carrying health risks—CAD the leading cause of death in industrialized populations—have persisted in human populations. A study by Rodriguez *et al.* [3], found evidence for multiple variants in genes related to ageing that exhibited antagonistic pleiotropic effects. They found higher risk allele frequencies with large effect sizes for late-onset diseases (relative to early-onset diseases) and an excess of variants with antagonistic effects expressed through early and late life diseases. There also exists other recent tangible evidence of antagonistic pleiotropy in specific human genes.

*SPATA31* gene has been found under strong positive genomic selection. Long-lived individuals carry fewer *SPATA31* copy numbers [4]. On the other hand, its overexpression in fibroblast cells leads to premature senescence, this being the case in people having multiple copies of the gene. During human evolution, more copies of this gene have likely been favored since this protein is important in sensing and repairing UV-induced DNA damage. Unfortunately, the cost is cell senescence and premature aging.

*GDF5* gene encodes for a protein important in joints and long bone development. Many people in Europe and Asia have a common variant in *GROW1*, an enhancer of the gene that causes reduced expression of *GDF5*. This common variant has been associated with decreased height and has been selected in people living in northern regions, since shorter stature is beneficial in cold environments [5]. This variant is found on a haplotype associated with elderly arthritis.

Another example of an allele that is associated with age-related degenerative disease, but may offer some advantage in younger ages, is ε4 of *APOE* gene. This allele is well known for Alzheimer’s disease predisposition after 60 years. Studies exist suggesting cognitive benefits of this risk allele at earlier ages, as young ε4 carriers have been shown to outperform non-carriers in measures of general cognitive ability, memory, attention, cognitive control allocation, verbal fluency, mental arithmetic performance and other brain functions [6 and references therein].

A study by Voskarides 2018 [7] found that genetic variants that have been selected for regarding the ability to live in extreme environmental conditions (cold and high-altitude adaptation), possibly also increase cancer risk at older ages. Interestingly, most of these variants are found on Tumor Suppressor Genes. It seems that reduced apoptosis potential in people living under demanding or toxic conditions make their cells more resistant in those environments. On the other hand, apoptosis resistance favors carcinogenesis progression in older ages. Inuit and Alaskan Indians, living at the colder regions of our planet, today have the highest cancer rates worldwide. Additionally, a statistically significant percentage of Inuit’s and Alaskan Indians’ genes under selection are Tumor Suppressor Genes [7].

These studies collectively demonstrate excellent candidate genes bearing the hallmarks of antagonistic pleiotropy. They appear to confer fitness advantages to human populations during the reproductive period, but also specifically increase risk of various common diseases that typically begin to manifest at ages when selection is weak. These genes would have been positively selected for if they improved survival and reproduction in ancestral environments, thereby increasing in frequency over time even if they increased morbidity in old ages. The manifestation of this tradeoff is conflated by the fact that post-reproductive life expectancy of modern humans well exceeds that of our ancestors living a few thousand years ago where age-related diseases would not have been the leading cause of death and therefore such tradeoffs would have been minimal anyway.

Further progress in human studies will be crucial to validate recent candidate genes and identify others with such antagonistic pleiotropic effects. It appears that a large percentage of genetic predisposition to age related diseases may have been shaped by evolutionary mechanisms. It may be important that these are considered alongside traditional GWAS disease risk variants and also given their pleiotropy, carefully considered regarding potential non-target effects in drug design or gene therapy.

**Conflict of interest:** None declared.
